# Elimination of Hepatitis Viruses: Bangladesh Scenario

**DOI:** 10.5005/jp-journals-10018-1209

**Published:** 2017-05-05

**Authors:** Mamun Al Mahtab

**Affiliations:** Department of Hepatology, Bangabandhu Sheikh Mujib Medical University, Dhaka, Bangladesh

**Keywords:** Bangladesh, Elimination, Viral hepatitis.

## Abstract

The World Health Organization (WHO) has adopted targets unanimously at the World Health Assembly in July 2016, in Geneva, to significantly curtail hepatitis B and C viruses to near extinction by 2030. Preparations are now ongoing in all WHO member nations across the globe to reach this ambitious, but perhaps achievable target. In Bangladesh, hepatologists, nongovernmental organizations, civil society, and patients have joined hands with the government in this global fight against viral hepatitis.

**How to cite this article:** Mahtab MA. Elimination of Hepatitis Viruses: Bangladesh Scenario. Euroasian J Hepato-Gastroenterol 2017;7(1):40-42.

## INTRODUCTION

**Fig. 1:**
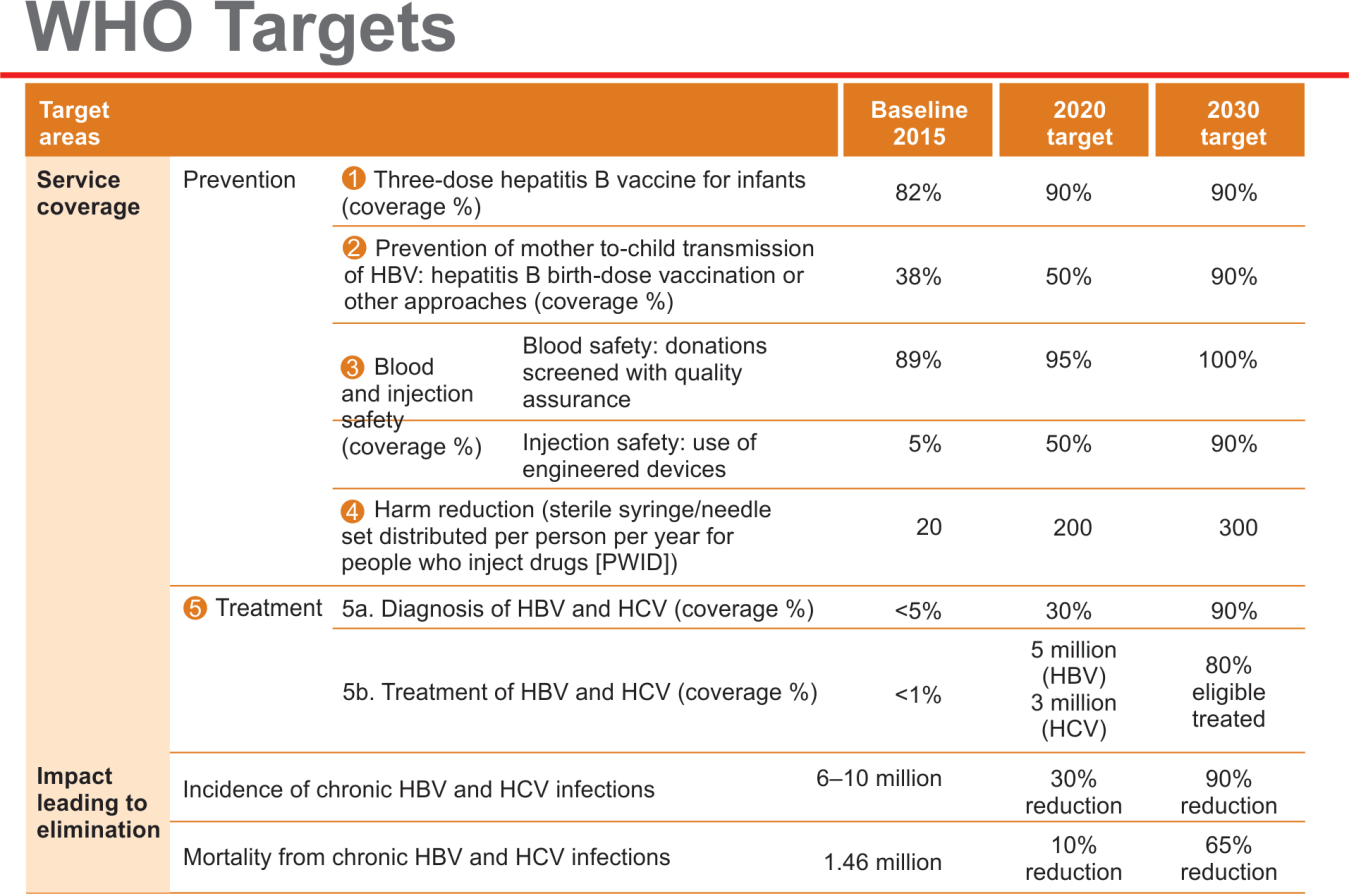
The WHO targets (retrieved from WHO Home Page)

The World Health Organization (WHO) in its last World Health Assembly unanimously adopted the resolution of "elimination" of viral hepatitis (Fig. 1). An account is provided on how elimination of viral hepatitis may be achieved in Bangladesh.

## BANGLADESH SCENARIO

In Bangladesh, we have excellent control programs for a range of communicable diseases, such as tuberculosis, malaria, dengue, and kala-azar, to name a few. However, there is no such program for hepatitis B virus (HBV) and hepatitis C virus (HCV) as of now. On the contrary, HBV-and HCV-related liver diseases incur a huge burden to our country’s economy and health care delivery system. It has been estimated that the prevalence of HBV and HCV in Bangladesh may be 5.4 and 0.84% respectively.^1,2^ Taking the figure into consideration, more than 10 million Bangladeshis are chronically infected with either of these two viruses. It has been estimated that HBV- and HCV-related liver diseases account for 8 to 12% admissions in the Medicine Departments of our tertiary hospitals, and are responsible for more than 20,000 deaths per annum, with hepatocellular carcinoma being the third most common cause of cancer deaths in Bangladesh, next only to deaths from cancers of lungs and stomach.^3^

Antivirals for HBV and HCV are still not in our essential drug list and there is no health insurance in Bangladesh. Treatment of HBV and HCV in Bangladesh is still, therefore, a private affair. However, the inspiring news is that with a vibrant pharmaceutical industry, Bangladeshi patients enjoy access to local generic versions of almost all antivirals for HBV and HCV. For HBV, in Bangladesh, we have locally produced generic lamivudine, adefovir, entecavir, and tenofovir. For HCV, the list includes generic pegylated interferon, ribavirin, and direct-acting antivirals (DAAs), such as sofosbuvir, ledipasvir, daclatasvir, and velpatasvir. In fact, the first generic sofosbuvir in the world was introduced in Bangladesh back in February 2015, making us the first country in the world to have a generic DAA. Not only so, recently, a local pharmaceutical company has introduced the first sofosbuvir-velpatasvir combination tablet. These have made antiviral drugs affordable to many in Bangladesh. Besides, tax waivers granted to DAAs by the Bangladesh government has further helped to bring down the costs. However, it is a reality that such treatments are still beyond the means of many of the Bangladeshi citizens.

We have a vibrant hepatology community in Bangladesh. Although not too many in number, they are contributing in this fight in different directions. The Association for the Study of Liver Diseases Bangladesh (ASLDB), the national body of Hepatologists of the country, is a sponsor member of the Asian Pacific Association for the Study of the Liver and South Asian Association for the Study of the Liver. The ASLDB has its own journal Bangladesh Journal of

Hepatology and our hepatologists are collaborating with the Euroasian Gastroenterological Association to regularly publish the Euroasian Journal of Hepato-Gastroenterology. At least six hepatology texts and handbooks have so far been edited by Bangladeshi hepatologists and published by international publishing companies. These have helped in the dissemination of knowledge to colleagues across disciplines both at home and abroad.

There has been major expansion in access to tertiary health care in Bangladesh over the last several years by the active support of the government of the People’s Republic of Bangladesh. Currently, there have been establishments of new superspeciality departments in different tertiary public hospitals. Hepatology has also benefited from this growth, and, currently, we have hepatology faculty positions in more than 20 public teaching hospitals across Bangladesh, thus facilitating access to quality, but affordable health care to our liver disease patients.

On the private side, there are several nongovernmental organizations (NGOs) active in this field. They are active in different arenas from creating awareness to providing access to treatment to collaborating with the government machinery to facilitate the battle against hepatitis viruses. In addition to some NGOs in this field, one new NGO, "Forum for the Study of the Liver Bangladesh (FSLB),"^4^ has become a voting member of the World Hepatitis Alliance (WHA). The FSLB runs its flagship project - Liver Care Research Centre - in the heart of Dhaka city, providing specialist consultation, investigations, antivirals, vaccination, and therapeutic interventions, at discounted rates to hepatitis patients. "Regional Hepatitis Summit 2016" that was joined by FSLB, Communicable Disease Control (CDC) of Directorate General of Health Services (DGHS), and WHA in Dhaka made an impact for the elimination of hepatitis in March 2016. The highlight of the summit was its versatile participant list that included hepatologists, media personalities, intellectuals, journalists, administrators, NGOs, donor agencies, representation from WHA, and regional organizations pursuing the common cause, and, most importantly, members of Patient’s Forum, a unique body comprising HBV- and HCV-infected patients, constituted under the auspices of FSLB. Strong government commitment for the cause was evident by the participation of two senior cabinet members at the summit, including the minister in-charge of the Ministry of Health and Family Welfare. The summit, thus, set the momentum for public-private partnership for "elimination."

The FSLB is now collaborating with the CDC of DGHS of the Bangladesh government to train physicians in the government health cadre on viral hepatitis. A training module has jointly been developed and, so far, more than 3,000 government physicians have been imparted training. Not only so, advocacy programs on viral hepatitis are being conducted regularly in different public and private teaching hospitals as well as public and private universities jointly.

The World Hepatitis Day (WHD) is now being observed in Bangladesh jointly by several NGOs like FSLB, Viral Hepatitis Foundation Bangladesh, and professional bodies like ASLDB and DGHS. Colorful rallies, theme songs, seminars, free screenings for HBV and HCV, etc., are highlights of observation of WHD in Bangladesh. This important issue has also attracted significant media attention, newspaper articles, and health shows in local electronic media, highlighting the issue that "elimination" is not infrequent in Bangladesh these days.

## CONCLUSION

With this background, although achieving the target of elimination by 2030 appears to be challenging in Bangladesh at this point, the ground is all set and appropriate interventions, if made timely through public-private partnership, for the target may well be achievable. It is to be noted with particular emphasis that the Bangladesh government has set an example in achieving most of the targets of the Millennium Development Goals.
